# Pharmacist-Administered Influenza Vaccination in Children and Corresponding Regulations

**DOI:** 10.3390/vaccines10091410

**Published:** 2022-08-28

**Authors:** Dana M. Gates, Steven A. Cohen, Kelly Orr, Aisling R. Caffrey

**Affiliations:** 1 College of Pharmacy, University of Rhode Island, Kingston, RI 02881, USA; 2 Department of Health Studies, College of Health Sciences, University of Rhode Island, Kingston, RI 02881, USA; 3 Infectious Disease Research Program, Providence Veterans Affairs Medical Center, Providence, RI 02908, USA; 4 Center of Innovation in Long-Term Support Services, Providence Veterans Affairs Medical Center, Providence, RI 02908, USA; 5 Department of Health Services Policy & Practice, Brown University School of Public Health, Providence, RI 02903, USA

**Keywords:** influenza vaccination, pharmacist-administered immunization, pediatric, immunization

## Abstract

In our retrospective cohort study, we evaluated trends in pharmacist-administered pediatric influenza vaccination rates in the United States and corresponding state-level pharmacist pediatric vaccination authorization models, including minimum age requirements, vaccination protocols, and/or prescription requirements. An administrative health claims database was used to capture influenza vaccinations in children less than 18 years old with 1 year of continuous enrollment and joinpoint regression was used to assess trends. Of the 3,937,376 pediatric influenza vaccinations identified over the study period, only 3.2% were pharmacist-administered (87.7% pediatrician offices, 2.3% convenience care clinics, 0.8% emergency care, and 6.0% other locations). Pharmacist-administered pediatric influenza vaccination was more commonly observed in older children (mean age 12.65 ± 3.26 years) and increased significantly by 19.2% annually over the study period (95% confidence interval 9.2%-30.2%, *p* < 0.05). The Northeast, with more restrictive authorization models, represented only 2.2% (n = 2816) of all pharmacist-administered pediatric influenza vaccinations. Utilization of pharmacist-administered pediatric influenza vaccination remains low. Providing children with greater access to vaccination with less restrictions may increase overall vaccination rates. Due to the COVID-19 pandemic and the Public Readiness and Emergency Preparedness Act, pharmacists will play a major role in vaccinating children.

## 1. Introduction

Although annual influenza vaccine is recommended by the Centers for Disease Control and Prevention (CDC) and the Advisory Committee on Immunization Practices (ACIP) for children 6 months and older [1,2,3,4,5,6], there is continued hesitancy towards influenza vaccination [7]. The CDC has consistently reported pediatric vaccination rates that are lower than the Healthy People 2020 target of 70% for children aged 6 months through 17 years [8,9], potentially due to barriers in access [10]. According to the National Immunization Survey (NIS), a national telephone survey conducted by the CDC to assess vaccination coverage among children (6 months through 17 years) [11], pediatric influenza vaccination rates over the past decade have increased from 51.0% in 2010–2011 to 63.8% in 2019–2020 [12].

While pediatric vaccines are often administered in pediatrician offices by nurses, nurse practitioners, medical assistants, and physician’s assistants, under the supervision of a physician, pharmacists have been approved influenza vaccinators in all 50 states since 2009 [13]. However, regulations enabling pharmacist-administered vaccination also carry restrictions, such as state-specific minimum age requirements and other regulatory limitations, including restricted lists of allowed vaccinations under these mandates [14,15,16]. Vaccination outside of pediatrician offices improves vaccine access through increased convenience, with expanded hours and without the need for an appointment [16]. Additionally, 93% of patients live within five miles of a pharmacy and most patients have positive experiences with their pharmacy [17,18].

To date, there are limited published estimates of pharmacist-administered pediatric influenza vaccination. According to NIS-Flu, pharmacy vaccination was reported for 5.5% of pediatric influenza vaccinations in the 2017–2018 influenza season (66.8% doctor’s offices, 16.2% clinics and health centers, 4.3% hospitals, 3.8% schools. 1.9% health departments, and 1.5% other setting) [19]. Due to the limitations of NIS-Flu, including overestimation of pediatric influenza vaccination and other biases due to the nature of survey methods [11], it is unclear whether these estimates of pharmacist-administered vaccination are accurate. Further, state-level variations in pharmacist-administered pediatric influenza vaccination remain unknown, as does the influence of state-level pharmacist vaccination authorization models.

As such, the objective of this study was to evaluate state-level pharmacist pediatric vaccination authorization models alongside state-level pharmacist-administered pediatric influenza vaccination rates in an administrative health claims database and assess time trends in pharmacist-administered pediatric influenza vaccination.

## 2. Materials and Methods

The distribution of vaccine providers among vaccinated children using an administrative health claims database with enrollees from all 50 states and the District of Columbia, the Optum Clinformatics^®^ Data Mart (OptumInsight, Eden Prairie, MN, USA) was assessed. Using a retrospective cohort study design, pediatric influenza vaccinations by vaccine provider setting and corresponding variations in patient characteristics were evaluated. The study population included children with an influenza vaccination record between 1 July 2010 to 30 June 2017 identified from Current Procedural Terminology, 4th Edition (CPT^®^) procedure codes found in Supplementary Table S1 and under 18 years of age at the time of the influenza vaccination claim. Vaccinations were assigned to influenza seasons, where time was divided into 12-month periods from 1 July through 30 June of the following year [20]. For each season, pediatric patients with full continuous enrollment for the 12 months of that season and with at least one influenza CPT^®^ code, were for selected for inclusion [20]. Only the first influenza vaccination within a season was included, while patients with missing year of birth, state, or sex were excluded.

Vaccine provider setting was categorized as pediatrician offices, which included family medicine and primary care settings, pharmacist-administered, convenience care clinic, emergency care, and other, which included locations such as schools or other immunization centers. Of note, pharmacy-based vaccination did not include healthcare clinics located in retail pharmacies (e.g., CVS MinuteClinic) as those were categorized under convenience care. Within each vaccine provider setting, we assessed the distribution of age and age group, sex, region, and pharmacist-authorization minimum age restrictions. Both mean and median age were assessed, as well as distribution by age groups per the NIS-Flu categorization of 6–23 months, 2–4 years, 5–12 years, and 13–17 years [19]. States were grouped into regions per the 2010 United States Census Bureau of Regions and Division [21] categorizations, as regional vaccination coverage can vary [22].

A grey literature search was performed to identify state-level regulations regarding pharmacist-authority to administer influenza vaccines in children. Grey literature search methods consisted of a Google search of key terms, including “pharmacist administered pediatric influenza vaccination” and “minimum age restrictions”. Search results were assessed for state-level regulations. One document was selected for inclusion after full-text review, authored by the American Pharmacists Association (APhA) and the National Alliance of State Pharmacy Associations (NASPA), due to the corresponding timeframe of the document and the study data [14]. Minimum age restrictions were assessed for all authorization models within each state.

The distribution of influenza vaccine provider setting among vaccinated children, and calculated pharmacist-administered pediatric influenza vaccination rates for each state (the proportion of vaccinated children with pharmacy provider setting within each state) was assessed. Pharmacist-administered pediatric influenza vaccination rates were then averaged among states with corresponding authorization models and minimum age requirements.

### Statistical Analysis

Demographic characteristics of vaccinated children in each provider setting were compared with the pediatrician office setting using chi-square tests, t-test, or Wilcoxon rank sum test, as appropriate, in SAS (version 9.4, SAS Institute Inc., Cary, NC, USA). Time trends in pediatric vaccination were assessed by vaccination provider setting with joinpoint regression, over the seven vaccination seasons with each season being an equally weighted interval. Average annual percent change (AAPC) and corresponding 95% confidence intervals by vaccination provider setting were calculated using the Joinpoint Regression Program, version 4.7.0.0 (National Cancer Institute, Bethesda, MD, USA). Statistical significance was considered *p* < 0.05.

## 3. Results

### 3.1. Pharmacist-Administered Pediatric Influenza Vaccination Rates

This study included 3,937,376 influenza vaccinations among children over seven influenza seasons, from 2010 to 2017 (Table 1). Of the total influenza vaccinations, most occurred in pediatrician offices (87.7%, n = 3,451,658), followed by pharmacist-administered (3.2%, n = 125,709), convenience care (2.3%, n = 90,304), and emergency care (0.8%, n = 32,499).

**Figure 1:**
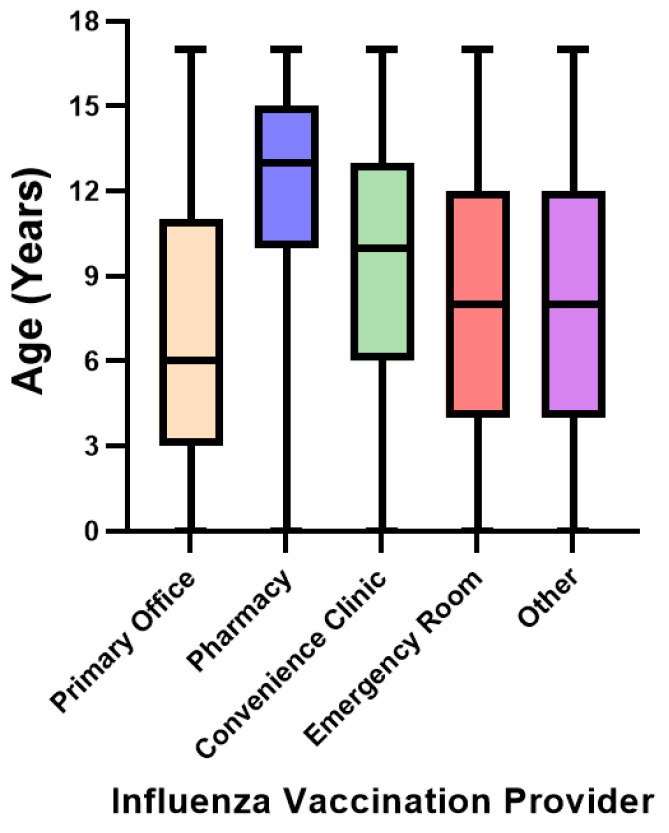
Median age of pediatric influenza vaccination by vaccine provider setting.

Mean and median ages (Table 1 and Figure 1) were highest among those vaccinated by a pharmacist (mean age 12.7 ± 3.3 years), followed by convenience care (mean 9.7 ± 4.2 years), other locations (mean 8.4 ± 4.9 years), emergency care (mean 8.2 ± 4.6 years), and youngest among those vaccinated in pediatrician offices (mean 7.0 ± 4.8 years). Within the pharmacy setting, 98.9% (n = 124,308) of influenza vaccinations were among children 5–17 years of age (56.3%, n = 70,806 in 13–17-year age group). In comparison, only 16.9% (n = 582,152) of children that were vaccinated in a pediatrician office were 13–17 years old.

Compared to the pediatrician office setting, the distribution of pediatric influenza vaccinations by sex differed significantly for the pharmacy (*p* = 0.004) and convenience care (*p* < 0.0001) settings. Influenza immunization of males was marginally higher than females in all settings, except for convenience care.

Statistically significant differences were observed in the distribution of geographic region when comparing pediatrician office to the other vaccine provider settings. Most vaccinations occurred in the South, for vaccination in the pediatrician office setting (39.5%), pharmacy (45.0%), and convenience care (53.0%), whereas most vaccinations in emergency care (50.0%) and other settings (36.7%) occurred in the West. Of all pharmacist-administered pediatric influenza vaccinations, only 2.2% (n = 2816) occurred in the Northeast, as Connecticut, Massachusetts, New York, and Vermont did not allow pharmacist-administered pediatric influenza vaccination.

**Figure 2:**
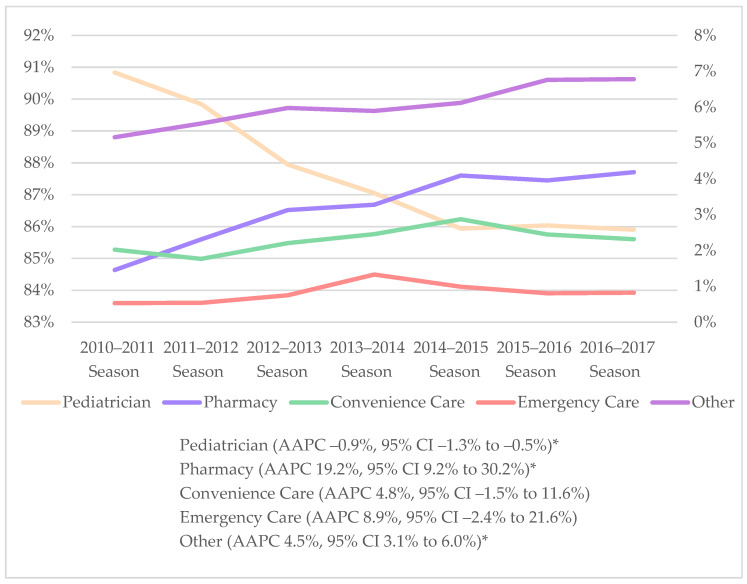
Trends in pediatric influenza vaccination by vaccine provider setting, 2010–2017. Note: AAPC, average annual percent change; CI, confidence interval. The Joinpoint Regression Program was used to calculate average annual percent change (AAPC) and 95% CI. * Significant trend at *p* < 0.05. Refer to left *y*-axis for influenza vaccination in pediatrician offices. Refer to right *y*-axis for all other influenza vaccine provider settings.

Over the study period, the average annual percent change in pharmacist-administered pediatric influenza vaccination increased significantly by 19.2% (95% CI 9.2 to 30.2, *p* < 0.05), from 1.5% in the 2010–2011 season to 4.2% in the 2016–2017 season (Figure 2). During the same period, the average annual percent change in pediatric influenza vaccination in pediatrician office decreased significantly by 0.9% (95% CI −1.3 to −0.5, *p* < 0.05).

### 3.2. Pharmacist-Administered Pediatric Influenza Vaccination Authorization Models

In the United States, the legislature of each state grants immunization authority to pharmacists under an approved scope of practice. There are three main state-level authorization models under which pharmacists can administer vaccinations (Table 2): prescription from physician provider, vaccination protocol, or pharmacist independent authority (neither physician prescription nor protocol required). A vaccination protocol specifies which vaccines can be administered under the protocol and vaccine administration procedures, including age limitations, usually agreed upon between state-level pharmacist groups, state-level physician groups, and the state public health department. Under a vaccination protocol, no prescription is required. In an independent authority model, pharmacists have authority to prescribe and administer vaccines without a protocol or physician prescription [23].

**Figure 3:**
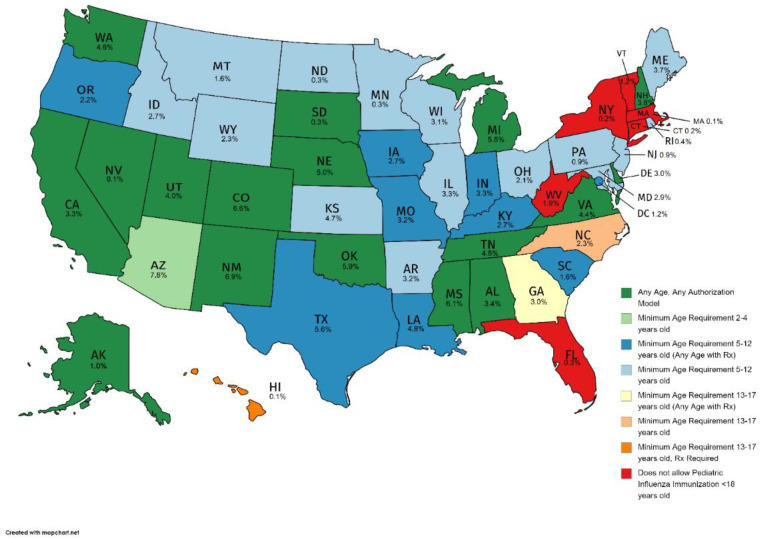
Pharmacist-administered pediatric influenza vaccination minimum age restrictions (as of July 2016) and pharmacist-administered pediatric influenza vaccination rates by state, 2010–2017. Note: Utilized youngest age of least restrictive authorization model for states with multiple authorization models.

Regardless of pharmacist authorization models (Table 2), the minimum age requirements allowing pharmacists to vaccinate against influenza vary by state (Figure 3). Arizona, where pharmacists are authorized to administer influenza vaccinations under two authorization models, the least restrictive being pharmacist independent authority beginning at 3 years of age, had the highest percent of children vaccinated by a pharmacist at 7.8% (Figure 3). Following Arizona were states where there is no age limit; New Mexico (6.9%), Colorado (6.6%), Nevada (6.1%), Mississippi (6.1%), Oklahoma (5.9%), Michigan (5.5%), and Nebraska (5.0%). The pharmacist-administered influenza vaccination rate in Texas was 5.6%, which allows pharmacist-administered influenza vaccination for children 7 years and older under a protocol and at any age with a prescription. Hawaii had the lowest pharmacist-administered influenza vaccination (0.1%), as the mandate only allows pharmacist-administered influenza vaccination in children 14 years and older with a physician prescription. As of July 2016 [14], and prior to the Public Readiness and Emergency Preparedness Act [24], six states did not allow pharmacist-administered influenza vaccination for children of any age, including Connecticut, Florida, Massachusetts, New York, Vermont, and West Virginia.

## 4. Discussion

To our knowledge, this is the first study to evaluate state-level pharmacy-based immunization rates among a commercially insured pediatric population in the United States and corresponding state-level authorization models. The majority of pediatric influenza vaccinations were found to be administered in pediatrician offices at 87.7%. This study found that over seven influenza seasons from 2010–2017, only 3.2% of all influenza vaccinations occurred in a pharmacy setting. However, also during this time, administration of pediatric influenza vaccines by pharmacists increased significantly by 19.2% each year. Pharmacist-administered influenza vaccination was more common among older children (mean age, 12.65 ± 3.26) and where administration laws have less restrictive age limitations around pharmacist-administered pediatric vaccination. States with more restrictions and older minimum age requirements, had lower rates of pharmacist-administered influenza immunization.

According to NIS-Flu data for the 2017–2018 influenza season, 5.5% of all children ages 6 months to 17 years old were immunized in a pharmacy setting, and 66.8% of children were vaccinated in their doctor’s office [19], while 28.3% of adults were vaccinated in a pharmacy and 34.7% in physicians’ offices [25]. NIS-Flu is a national dual landline and cellular list-assisted random-digit-dialed telephone survey of households. This self-reported survey data is subject to recall bias and non-response bias from low response rates [11]. The findings presented from this cohort study were from administrative claims data from a commercially insured population with records of actual influenza vaccine administrations by provider setting and are not subject to survey data biases.

As also reported by NIS-Flu data, influenza vaccine setting varied by age group; for example children less than 5 years old utilized pediatrician offices more frequently than other settings and children 13–17 years old utilized a pharmacy setting more frequently [19]. This is noted in the findings presented here as well, as 98.9% of children that were vaccinated in a pharmacy were 5–17 years old, with 56.3% in the 13–17-year age group versus 16.9% in the 13–17 years old age group for pediatrician offices. Administration of annual influenza vaccine typically occurs around the same time every year, in late fall or early winter, which may not align with a child’s annual routine examination. As children get older, they are less likely to receive the influenza vaccine in general. For example, in the 2015–2016 influenza season, vaccination coverage rates within age groups were as follows; 6 months-23 months 75.3%, 2–4 years 66.8%, 5–12 years 61.8%, and 13–17 years 46.8% [25]. This has been a consistent trend since 2010 according to NIS-Flu data [19]. This may be due to a decrease in the number of physician visits as children get older, and therefore increased interest in vaccination outside of that setting, such as a pharmacy, would provide.

A main objective of pharmacist-administered influenza immunization is to expand access to vaccination through increased accessibility, convenience, extended hours, and widespread locations [8] for patients who find it difficult to reach traditional healthcare services, which could in turn increase vaccination coverage for all ages [18]. Pharmacies are open for longer hours during the week and are also available on weekends and holidays. A study from the 2011–2012 influenza season estimated that over 30% of all adult vaccinations administered at a Walgreens pharmacy chain were during evenings, weekends, or holidays [26]. Another important role pharmacists play is through vaccine advocacy [27]. A study conducted among adults showed that pharmacists were more successful than traditional healthcare providers in administering vaccines to patients that were not current in their immunizations after performing an immunization needs assessment [8]. However, a scenario where pharmacists require a prescription from a healthcare provider to administer vaccines to patients, particularly in children, can be more costly and less efficient [23].

Increasing influenza vaccination among children is critical to controlling influenza outbreaks. It has been found that vaccinating children, in addition to protecting other children, protects the entire community by limiting the transmission of the virus, in particular to vulnerable populations such as the elderly and immunocompromised [28]. Expansion of the ability for pharmacists to administer vaccines and increase vaccination rates among children, for influenza and other epidemics, is critical for public health.

As of August 2020, pharmacists in all states have been authorized by the U.S. Health and Human Services Department (HHS) to administer any FDA authorized vaccines to children aged 3 to 18 under the “Third Amendment to Declaration Under the Public Readiness and Emergency Preparedness Act for Medical Countermeasures Against COVID–19” (PREP Act) for the duration of the COVID-19 pandemic [24]. Due to the current COVID-19 pandemic, there has been an alarming decline in routine pediatric vaccines due to decreased pediatric physician visits and changes in healthcare access (such as closed offices or limited hours, and corresponding increases in virtual visits) [24,29]. Data collected from the Vaccines for Children program, a national program that provides vaccines to approximately 50% of children in the United States [29], showed that physicians participating in the program ordered approximately 2.5 million fewer non-influenza routine vaccines and 250,000 less measles-containing vaccines, and the decline was more prominent in children 2–18 years old [29,30]. In the declaration, pharmacists are recognized as being well positioned to expand access to all childhood vaccinations, which can help combat the public health threat of declining vaccination rates [24].

As this amendment is recent and parents may not be aware of this change, it is unclear to what extent parents will elect to have their children vaccinated by a pharmacist. National and community-level campaigns to raise awareness about the availability and convenience of pediatric vaccination at local pharmacies may increase uptake. Future efforts should monitor uptake of pharmacist-administered pediatric vaccination to determine utilization rates, and whether increased vaccination by pharmacists compensates for reductions in pediatrician office vaccinations. It is possible that societal vaccination practices may shift as a result of this expansion in care, similar to changes observed in satisfaction with and therefore preference in some cases for virtual healthcare [31]. It remains unknown how long pharmacist-administered vaccination authorization will remain under the PREP Act. Studying its long-term impacts will be important for advocacy efforts of continued expanded access to vaccination for children in the United States at their local pharmacies, particularly if pharmacists become the primary vaccine provider for pediatric COVID-19 vaccines.

### Limitations

The population studied here was limited to pediatric patients from one administrative health claims database within the U.S., compiled from vaccination procedure codes. As such, this study did not assess influenza vaccination by provider setting among pediatric patients with Medicaid, which is estimated to include approximately 30 million children nationally, or other private health plans [32], and distributions by vaccine provider may vary in these populations. Missing from this cohort are influenza vaccinations that were not processed through health insurance, such as through free public health clinics. Due to the nature of the database, age was calculated using the date of vaccination and the patient’s year of birth. In patients that are vaccinated in early fall and have birthdays occurring later in the year, the patient would be considered one year older if they were vaccinated before their birthday. The study relies on the accuracy and consistency of provider categorization in the source data to indicate vaccine administration setting. Additionally, the study population available from this database is now several years old, and future research should evaluate whether these trends are still observed in more current populations. Vaccinations in other locations, including non-specific locations were uncommon, encompassing 6.0% of pediatric influenza vaccination in this study population. Overall pediatric influenza vaccination rates vary by state [19], as did the proportion of children within this dataset of the total state pediatric population, which we did not assess in this study. Race and ethnicity were not available from the data source, and therefore, could not be assessed. Pharmacist authorization models were assessed as of July 2016, and therefore changes in authorization models may have occurred over the 7-year period when pediatric influenza vaccination administrative claims were assessed.

## 5. Conclusions

Although pharmacist-administered influenza vaccination has been allowed in all 50 states for over 10 years [13], various state-level restrictions on pharmacist-administered pediatric influenza vaccination, such as protocols, prescription requirements, and minimum age restrictions, impact uptake of pharmacist-administered pediatric influenza vaccination, as we observed the lowest pharmacist-administered influenza vaccination rates in states with the most restrictive laws. Of the nearly 4 million, pediatric influenza vaccinations included in this study, only 3.2% were pharmacist-administered. However, utilization of pharmacists as influenza vaccinators of children increased significantly by 19.2% each year over our 7-year study period, with most pharmacist-administered influenza vaccinations being administered to older children (mean age, 12.65 ± 3.26). Providing children with greater access to vaccinations with fewer restrictions could help increase influenza vaccination rates to get closer to the Healthy People 2020 target of at least 70% for children [9]. Additionally, during the ongoing COVID-19 pandemic, pharmacists will be instrumental for overcoming the profound decrease in all pediatric vaccination rates observed during the pandemic [24]. Future research should evaluate uptake of pharmacist-administered pediatric vaccination for influenza, other childhood vaccines, and pediatric COVID-19 vaccination when such data become available.

## Figures and Tables

**Table 1:** Pediatric influenza vaccinations by vaccine provider setting.

Demographic Characteristics and Authorization Models	Pediatrician Office	Pharmacy	Convenience Clinic	Emergency Care	Other
	n = 3,451,658 (87.7%)	n = 125,709 (3.2%)	n = 90,304 (2.3%)	n = 32,499 (0.8%)	n = 237,206 (6.0%)
**Age** (Mean ± SD)	7.04 ± 4.81	12.65 ± 3.26 *	9.73 ± 4.23 *	8.21 ± 4.64 *	8.37 ± 4.90 *
**Age Group** ^†^					
6 months–2 years	768,750 (22.3%)	87 (0.1%) *	2784 (3.1%) *	4092 (12.6%) *	35,227 (14.9%) *
2–4 years	520,856 (15.1%)	1314 (1.1%) *	8775 (9.7%) *	4221 (13.0%) *	28,300 (11.9%) *
5–12 years	1,579,900 (45.8%)	53,502 (42.6%) *	51,919 (57.5%) *	17,178 (52.9%) *	115,465 (48.7%) *
13–17 years	582,152 (16.9%)	70,806 (56.3%) *	26,826 (29.7%) *	7008 (21.6%) *	58,214 (24.5%) *
**Sex** ^†^					
Female	1,687,410 (48.9%)	62,095 (49.4%) *	45,513 (50.4%) *	15,959 (49.1%)	116,650 (49.2%) *
Male	1,764,248 (51.1%)	63,614 (50.6%) *	44,791 (49.6%) *	16,540 (50.9%)	120,556 (50.8%) *
**Region** ^†^					
Midwest ^1^	969,480 (28.1%)	28,548 (22.7%) *	24,134 (26.7%) *	6178 (19.0%) *	82,370 (34.7%) *
Northeast ^2^	486,435 (14.1%)	2816 (2.2%) *	6934 (7.7%) *	1775 (5.5%) *	17,536 (7.4%) *
South ^3^	1,361,550 (39.5%)	56,517 (45.0%) *	47,898 (53.0%) *	8288 (25.5%) *	50,333 (21.2%) *
West ^4^	634,193 (18.4%)	37,828 (30.1%) *	11,338 (12.6%) *	16,258 (50.0%) *	86,967 (36.7%) *
**State-Level Pharmacist Authorization Model, Age Restrictions** ^†^					
No age restriction	822121 (23.8%)	46238 (36.8%) *	14429 (16.0%) *	17369 (53.4%) *	94696 (39.9%) *
Min age 2–4 years old	80514 (2.3%)	7509 (6.0%) *	4899 (5.4%) *	85 (0.3%) *	3119 (1.3%) *
Min age 5–12 years old, Any age with Rx	784427 (22.7%)	39765 (31.6%) *	21981 (24.3%) *	3477 (10.7%) *	33708 (14.2%) *
Min age 5–12 years old	1031281 (29.9%)	22207 (17.7%) *	23692 (26.2%) *	7701 (23.7%) *	79558 (33.5%) *
Min age 13–17 years, Any age with Rx	175394 (5.1%)	6099 (4.9%) *	8103 (9.0%) *	863 (2.7%) *	10549 (4.5%) *
Min age 13–17 years old ^5^	103572 (3.0%)	2652 (2.1%) *	5086 (5.6%) *	363 (1.1%) *	3003 (1.3%) *
Min 18 years old	454349 (13.2%)	1239 (1.0%) *	12114 (13.4%) *	2641 (8.1%) *	12573 (5.3%) *

^†^ No. (%). * *p* < 0.05. ^1^ Ohio, Indiana, Michigan, Illinois, Wisconsin, Missouri, Iowa, Minnesota, North Dakota, South Dakota, Nebraska, Kansas. ^2^ Maine, New Hampshire, Vermont, Massachusetts, Rhode Island, Connecticut, New York, New Jersey, Pennsylvania. ^3^ West Virginia, District of Columbia (DC), Virginia, North Carolina, Kentucky, Maryland, Delaware, Tennessee, South Carolina, Georgia, Florida, Alabama, Mississippi, Arkansas, Louisiana, Oklahoma, Texas. ^4^ Montana, Wyoming, Colorado, Utah, Idaho, Nevada, Washington, Oregon, California, Alaska, Hawaii, New Mexico, Arizona. ^5^ Includes Hawaii, where a prescription is required for pharmacist-administered influenza vaccination.

**Table 2:** Pharmacist-administered pediatric influenza vaccination authorization models and average pharmacist-administered pediatric influenza vaccination rates, 2010–2017.

Authorization Model	Patient Age limitations	State(s)	Average Pharmacist-Administered Pediatric Influenza Vaccination Rates, by Authorization Model and age RESTRICTION	Average Pharmacist-Administered Pediatric Influenza Vaccination Rates, by Authorization Model
Pharmacist independent authority ^1^	Any Age	Alaska New Hampshire New Mexico	4.0%	3.3%
	6 months *	Virginia ^1,3^		
	3	Arizona ^1,2^ California ^1,2^	5.6%	
	6	Idaho	2.7%	
	7	Louisiana ^1,3^ Maine Oregon ^1,3^ Wyoming	3.3%	
	9	Maryland	2.9%	
	12	Montana New Jersey ^1,3^ South Carolina ^1,3^	1.4%	
Protocol ^2^	Any Age	Alabama California ^1,2^ Colorado Delaware Michigan Mississippi Nebraska Nevada Oklahoma South Dakota Tennessee Utah Washington	4.5%	3.5%
	5	North Dakota	0.3%	
	6	Arizona ^1,2^ Iowa ^2,3^ Kansas Minnesota Wisconsin	3.7%	
	7	Arkansas Ohio Texas ^2,3^	3.6%	
	9	Kentucky ^2,3^ Pennsylvania Rhode Island	1.3%	
	10	Illinois	3.3%	
	11	Indiana ^2,3^	3.3%	
	12	District of Columbia ^2,3^ Missouri ^2,3^	2.2%	
	13	Georgia ^2,3^	3.0%	
	14	North Carolina	2.3%	
Prescription ^3^	Any Age	District of Columbia ^2,3^ Georgia ^2,3^ Indiana ^2,3^ Iowa ^2,3^ Kentucky ^2,3^ Louisiana ^1,3^ Missouri ^2,3^ Oregon ^1,3^ South Carolina ^1,3^ Texas ^2,3^ Virginia ^1,3^	3.2%	2.8%
	6	New Jersey ^1,3^	0.9%	
	14	Hawaii	0.1%	

Note: Pharmacist-administered pediatric influenza vaccination authorization models as of July 2016 [14]. Authorization models are not mutually exclusive for each state, as states may have different authorization models for different age groups. * Influenza vaccine is recommended in children 6 months of age and older. ^1^ Pharmacists have authority to administer vaccines without a protocol or prescription. ^2^ Vaccination protocol specifies vaccine administration procedures usually agreed upon between state-level pharmacist and physician groups and the state public health department. ^3^ Prescription from physician provider is required prior to administration of vaccine by pharmacist.

## Data Availability

Due to the data use agreement in place with the data source, study data cannot be shared.

## Supplementary Materials

The following supporting information can be downloaded at: https://www.mdpi.com/article/10.3390/vaccines10091410/s1, Table S1. Current Procedural Terminology (CPT) codes for influenza vaccination.
