# Supplementary material for: Pharmacist-Administered Influenza Vaccination in Children and Corresponding Regulations
Source: Vaccines (Basel). 2022 Aug 28;10(9):1410. doi: 10.3390/vaccines10091410 (PMC9505660; doi:10.3390/vaccines10091410)
Supplement: Supplementary file 1 [file vaccines-10-01410-s001.zip › vaccines-1876936-supplementary.pdf]

Supplementary Table S1. Current Procedural Terminology (CPT) codes for influenza vaccination

CPT Code	Description
9952	PROPH VACCINATION AGAINST FLU
90470	H1N1 IMMUNIZATION, IM AND INTRANASAL
90630	FLU VACC IIV4 NO PRESERV ID
90653	IIV ADJUVANT VACCINE IM
90654	FLU VACC IIV3 NO PRESERV ID
90655	IIV3 VACC NO PRSV 0.25 ML IM
90656	IIV3 VACC NO PRSV 0.5 ML IM
90657	IIV3 VACCINE SPLT 0.25 ML IM
90658	IIV3 VACCINE SPLT 0.5 ML IM
90659	FLU VACCINE WHOLE IM
90660	LAIV3 VACCINE INTRANASAL
90661	CCIIV3 VAC NO PRSV 0.5 ML IM
90662	IIV NO PRSV INCREASED AG IM
90663	FLU VACC PANDEMIC H1N1
90664	LAIV VACC PANDEMIC INTRANASAL
90666	FLU VAC PANDEM PRSRV FREE IM
90667	IIV VACC PANDEMIC ADJUVT IM
90668	IIV VACCINE PANDEMIC IM
90672	LAIV4 VACCINE INTRANASAL
90673	RIV3 VACCINE NO PRESERV IM
90674	CCIV4 VAC NO PRSV 0.5 ML IM
90682	RIV4 VACC RECOMBINANT DNA PRSRV ANTIBIO FREE IM
90685	IIV4 VACC NO PRSV 0.25 ML IM
90686	IIV4 VACC NO PRSV 0.5 ML IM
90687	IIV4 VACCINE SPLT 0.25 ML IM
90688	IIV4 VACCINE SPLT 0.5 ML IM
90689	IIV4 VACC INACTIVATED, ADJUVANTED, PRSV FREE 0.25 ML IM
90724	INFLUENZA IMMUNIZATION
90737	INFLUENZA B IMMUNIZATION
90756	IIV4 VACC INACTIVATED ABX FREE 0.5 ML
3E01340	INTRO FLU VACCINE SUBQ TISSUE PC
4035F	INFLUENZA IMM REC
4037F	INFLUENZA IMM ORDER/ADMIN
G0008	ADMINISTRATION INFLUENZA VIRUS VACC
G8108	PT DOC RECV FLU VACC DUR FLU SEASON
G9141	INFLUENZA A H1N1 IMMUNIZATION ADMIN

G9142	INFLUENZA A H1N1 VACCINE ANY ROUTE
Q0034	INFLUENZA VACCI; MEDICAR
Q2033	INF VACC RECOMB HEMAGGLUTININ AG IM
Q2034	FLU VIRUS VAC SPLIT VRS IM AGRIFLU
Q2035	FLU VACC SPLIT 3 YRS & > IM AFLURIA
Q2036	FLU VACC SPLIT 3 YR & > IM FLULAVAL
Q2037	FLU VACC SPLIT 3 YR & > IM FLUVIRIN
Q2038	FLU VACC SPLIT 3 YRS & > IM FLUZONE
Q2039	INFLUENZA VIRUS VACCINE NOS
